# Contributions to the systematics of New World macro-moths V

**DOI:** 10.3897/zookeys.421.8050

**Published:** 2014-06-27

**Authors:** B. Christian Schmidt, J. Donald Lafontaine

**Affiliations:** 1 Canadian Food Inspection Agency, Canadian National Collection of Insects, Arachnids and Nematodes, K.W.Neatby Bldg., 960 Carling Ave., Ottawa, ON, Canada K1A 0C6; 2 Canadian National Collection of Insects,Arachnids, and Nematodes, Biodiversity Program, Agriculture and Agri-Food Canada, KW NeatbyBldg.,C.E.F., Ottawa, Ontario, Canada K1A 0C6

With the fifth installment of the “Contributions to the systematics of New World macro-moths” series, initiated in 2009 (Schmidt and Lafontaine 2009), systematics of taxa in the Geometridae, Notodontidae, Erebidae and Noctuidae are addressed. Geographic coverage is approximately equal between the Nearctic and Neotropic realms.

Despite the relatively advanced taxonomic knowledge of the North American macro-moth fauna, surprising discoveries continue – one new genus and species of Noctuidae is described from eastern North America (*Cherokeea* Quinter & Sullivan, gen. n.; *Cherookeea attakullakulla* Sullivan & Quinter, sp. n.), and two genera and four species, also Noctuidae, from western North America (*Chloronycta* Schmidt & Anweiler, gen. n., *Nudorthodes* Lafontaine, Walsh & Ferris, gen. n., *Nudorthodes molino* Lafontaine, Walsh & Ferris, sp. n., *Protorthodes ustulata* Lafontaine, Walsh & Ferris, sp. n., *Protorthodes texicana* Lafontaine, sp. n., *Protorthodes mexicana* Lafontaine, sp. n.). Four new generic combinations are proposed: *Chloronycta tybo* (Barnes), *Acronicta fallax* (Herrich-Schäffer), comb. n., *Nudorthodes texana* (Smith, 1900), comb. n., and *Nudorthodes variabilis* (Barnes & McDunnough, 1912), comb. n. Six previously recognized species are revised in status to subspecies: *Raphia frater abrupta* Grote, stat. n., *Raphia frater coloradensis* Putnam-Cramer, stat. rev., *Raphia frater piazzi* Hill, stat. n., and *Raphia frater elbea* Smith, stat. n. Ongoing updates and corrections to the North American Noctuoidea checklist (Lafontaine and Schmidt 2011, 2013) will be published in the upcoming volume VI of “Contributions”.

Eight new species are described from the Neotropics, all from Costa Rica: *Phyllodonta esperanza* Sullivan, sp. n., *Phyllodonta intermediata* Sullivan, sp. n., *Phyllodonta alajuela* Sullivan, sp. n. (Geometridae); *Symmerista luisdiegogomezi* Chacón, sp. n., *Symmerista aura* Chacón, sp. n., *Symmerista inbioi* Chacón, sp. n., *Symmerista minaei* Chacón, sp. n. and *Disphragis bifurcata* Sullivan & Pogue, sp. n. (Notodontidae). The status of *Disphragis hemicera* (Schaus, 1910), stat. rev. is raised to species and *Elymiotis tlotzin* (Schaus, 1892), comb. n. is transferred from *Symmerista*. The type material of Neotropical Arctiinae (Erebidae) described by C. G. Burmesiter and C. Berg is reviewed, resulting in the following changes: *Opharus picturata* (Burmeister, 1878), comb. n. (= *Opharus brunnea* Gaede, 1923: 7, syn. n.); *Hypocrisias fuscipennis* (Burmeister, 1878) (= *Phaegoptera jonesi* Schaus, 1894, syn. n.); *Leucanopsis infucata* (Berg, 1882), stat. rev.; *Paracles argentina* (Berg, 1877), sp. rev.; *Paracles uruguayensis* (Berg, 1886), sp. rev. A lectotype is designated for *Halesidota picturata* Burmeister, 1878, *Halesidota cancellata* Burmeister, 1878 *Halesidota cancellata* Burmeister, 1878.

Seventeen authors contributed eight manuscripts for this volume, and the continued interest and support of these contributors makes this series (Schmidt and Lafontaine 2009, 2010, 2011, 2013) possible. Authors interested in contributing to future editions of “Contributions …” are encouraged to contact us.
